# Supplementary material for: Senescence and entrenchment in evolution of amino acid sites
Source: Nat Commun. 2020 Sep 14;11:4603. doi: 10.1038/s41467-020-18366-z (PMC7490271; doi:10.1038/s41467-020-18366-z)
Supplement: Supplementary file 1 — Supplementary Information [file 41467_2020_18366_MOESM1_ESM.pdf]

Senescence and entrenchment in evolution of amino acid sites

Supplementary Note 1

Formal proof that random changes in SPFL decrease the fitnes of the current allele

Consider a locus under selection with N different alleles. These alleles differ in fitness that they confer, so that allele i confers fitness w_i. The vector of these fitness values, SPFL, can change instantaneously. At the time of such a change, each allele is, in general, ascribed a new fitness value w'_i, giving rise to a new SPFL. We assume that the effective population size is constant, so that the substitution rate between alleles i and j depends on the difference in their fitness $s_{ij} = w_i - w_j$.

Notation

τ_M	=	average time between substitutions
τ_L	=	average time between SPFL changes
N	=	number of possible alleles
a_i	=	probability that the considered position is occupied by the allele i
w_i	=	fitness of allele i at the considered position before the SPFL change
w'_i	=	fitness of allele i at the considered position after the SPFL change
$s_{ij} = w_i - w_j$	=	selection coefficient in favor of the allele i compared to the allele j at the considered position
$f_{ij} = f(s_{ij})$	=	probability of i to j substitution in a given position as a function of the selection coefficient.

Here we consider allele substitutions as a Markov process, and don't account for changes in allele frequencies within the population. The probability of an allele to be fixed monotonically increases with its fitness [1], so we assume that $f(s_{ij})$ is (strictly) monotonically increasing.

The sum of allele frequencies equals 1:

$$\sum_i^N a_i = 1\,. \tag{1}$$

The fitness vector is normalized so that the sum of fitness values for all alleles also equals 1:

$$\sum_i^N w_i = 1\,. \tag{2}$$

A change in SPFL may increase the fitness of the allele currently occupying an amino acid position, decrease it, or leave it unchanged. We shall prove that a random SPFL change will **on average** decrease the fitness of the current allele:

$$\beta = \langle \Delta w \rangle_{i,w'} = \langle w'_i - w_i \rangle_{i,w'} = \langle \sum_i (w'_i - w_i) a_i \rangle_{w'} < 0\,, \tag{3}$$

where the averaging is done over all alleles i and over all possible new SPFLs (or, analogously, over all new allele fitness values w').

1. Rare SPFL changes ($\tau_M \ll \tau_L$)

If the average time between SPFL changes is significantly larger that the average time between substitutions (i. e. $\tau_M \ll \tau_L$), the distribution of allele frequencies after a substitution reaches the equilibrium state, i.e. the allele

frequencies vector is the eigenvector for the substitution probability matrix:

$$a_i = \sum_k f_{ik} a_k \,. \tag{4}$$

Since the allele distribution reaches equilibrium after each SPFL change, the difference between frequencies of any two alleles depends only on the fitness vector:

$$a_i - a_j = \sum_k f_{ik} a_k - \sum_k f_{jk} a_k = \sum_k (f_{ik} - f_{jk}) a_k = \sum_k (f(w_i - w_k) - f(w_j - w_k)) a_, . \tag{5}$$

If w_i is larger than w_j, then $\forall k : w_i - w_k > w_j - w_k$. Since f is monotonically increasing, this implies that $f(w_i - w_k) > f(w_j - w_k)$. Therefore, since a_k can't be negative, the difference between a_i and a_j is also non-negative:

$$w_i > w_j \Rightarrow a_i > a_j \,. \tag{6}$$

Let's transform the equation 3 :

$$\beta = \langle \Delta w \rangle_{i,w'} = \langle \sum_i (w'_i - w_i) a_i \rangle_{w'} = \sum_i (\langle w'_i \rangle_{w'} - w_i) a_i \,. \tag{7}$$

1.1. The new SPFL is independent of the previous SPFL

First assume that the SPFL changes are random and not "biased" towards any alleles, meaning that all alleles have the same average fitness α across all the possible SPFLs. Then, based on the alleles fitness values normalization (equation 2),

$$\alpha = 1/N \,. \tag{8}$$

Replacing the average new fitness $\langle w'_i \rangle_{w'}$ in eqn. 7 with the value from eqn. 8,

$$\beta = \sum_i (\langle w'_i \rangle_{w'} - w_i) a_i = \sum_i (1/N - w_i) a_i \,. \tag{9}$$

The fitness values of the alleles should remain normalized after the SPFL change:

$$\sum_i^N w'_i = 1 \,. \tag{10}$$

The fitness of the allele i can be either less than $1/N$ (let's denote this set of alleles as N^-), greater than $1/N$ (N^+), or equal to $1/N$ (N^0). Then

$$\beta = \sum_{i \in N^+} (1/N - w_i) a_i + \sum_{i \in N^-} (1/N - w_i) a_i + \sum_{i \in N^0} (1/N - w_i) a_i = \sum_{i \in N^+} (1/N - w_i) a_i + \sum_{i \in N^-} (1/N - w_i) a_i \,. \tag{11}$$

Let's denote the allele with the smallest fitness greater than $1/N$ as i_1 and the allele with the largest fitness less than $1/N$ as i_2:

$$\begin{aligned} w_{i_1} &> 1/N, \\ \forall i \in N^+ : w_i &\geq w_{i_1} ; \\ w_{i_2} &< 1/N, \\ \forall i \in N^- : w_i &\leq w_{i_2} . \end{aligned} \tag{12}$$

Then, from equation 6,

$$\begin{aligned} \forall i \in N^+ : a_i &\geq a_{i_1} ; \\ \forall i \in N^- : a_i &\leq a_{i_2} ; \\ a_{i_1} &> a_{i_2} \end{aligned} \tag{13}$$

$$\sum_{i\in N^+}(1/N - w_i)a_i < \sum_{i\in N^+}(1/N - w_i)a_{i_1}$$
$$\sum_{i\in N^-}(1/N - w_i)a_i < \sum_{i\in N^-}(1/N - w_i)a_{i_2}\,. \tag{14}$$

Then we can estimate β from equation 11:

$$\beta = \sum_{i\in N^+}(1/N - w_i)a_i + \sum_{i\in N^-}(1/N - w_i)a_i < \sum_{i\in N^+}(1/N - w_i)a_{i_1} + \sum_{i\in N^-}(1/N - w_i)a_{i_2} =$$
$$a_{i_1}\sum_{i\in N^+}(1/N - w_i) + a_{i_2}\sum_{i\in N^-}(1/N - w_i)\,. \tag{15}$$

Since the fitness values of individual alleles are normalized,

$$\sum_{i\in N^+}(1/N - w_i) + \sum_{i\in N^-}(1/N - w_i) = \sum_{i}^{N}(1/N - w_i) = \sum_{i}^{N} w_i - N(1/N) = 1 - 1 = 0\,. \tag{16}$$

Let's denote

$$\sum_{i\in N^+}(1/N - w_i) = -\sum_{i\in N^-}(1/N - w_i) = \gamma < 0$$
$$\beta = \sum_{i}(1/N - w_i)a_i < a_{i1}\gamma - a_{i2}\gamma = (a_{i1} - a_{i2})\gamma < 0\,. \tag{17}$$

Therefore, the average allele fitness change after a random SPFL change $\langle \Delta w\rangle_{i,w'} = \beta < 0$.

1.2. The new SPFL is partially correlated with the old one

We shall prove that even if the fitness values after a SPFL change (w') are not completely random, but are correlated with the previous SPFL (w), the average change in fitness of the current alleles after a random SPFL change is still negative.

Let's assume that the new SPFL has a random component w^r contributing the fraction δ ($0 < \delta < 1$) of the SPFL, and the remainder $(1 - \delta)$ comes from the old SPFL:

$$w' = \delta w^r + (1 - \delta)w\,. \tag{18}$$

Equation 7 can then be transformed:

$$\beta = \langle\sum_i (w'_i - w_i)a_i\rangle_{w'} = \sum_i(\langle w'_i\rangle_{w'} - w_i)a_i = \sum_i\langle \delta w^r_i + (1-\delta)w_i\rangle_{w'}a_i - \sum_i w_i a_i =$$
$$\delta\sum_i\langle w^r_i\rangle_{w'}a_i + (1-\delta)\sum_i\langle w_i\rangle_{w'}a_i - \sum_i w_i a_i\,. \tag{19}$$

Since the old SPFL doesn't depend on the new SPFL, and $\langle w_i\rangle_{w'} = w_i$,

$$\beta = \delta(\sum_i\langle w^r_i\rangle_{w'}a_i - \sum_i w_i a_i) = \delta\sum_i(1/N - w_i)a_i\,. \tag{20}$$

Since $\delta > 0$ and, as we already proved, $\sum_i(1/N - w_i)a_i$ is negative (eqn. 17), β is also negative.

2. SPFL changes at rate comparable with the evolution rate ($\tau_M \approx \tau_L$)

If the SPFL changes are not so rare, the distribution of allele probabilities a_i after a substitution doesn't reach the equilibrium state, meaning that a_i does not necessary increase monotonically with the increase of w_i and that equation 6 is not applicable. However, if there is a correlation between w_i and a_i which is decreased by random changes in SPFL, such changes will still result in a decline in the average fitness of the current allele.

Let us represent the relationship between w_i and a_i as the ratio between the components of w_i and a_i vectors, i. e. covariance $cov(a, w)$. Based on eqn. 1 and eqn. 2,

$$cov(a,w) = \langle w_i a_i \rangle_i - \langle w_i \rangle_i \langle a_i \rangle_i = (1/N)\sum_i w_i a_i - ((1/N)\sum_i w_i)((1/N)\sum_i a_i) = (1/N)\sum_i w_i a_i - (1/N^2)\,. \tag{21}$$

The equation 3 can be transformed:

$$\beta = \langle \Delta w \rangle_{i,w'} = \langle w'_i - w_i \rangle_{i,w'} = \langle \sum_i (w'_i - w_i) a_i \rangle_{w'} = \langle \sum_i w'_i a_i \rangle_{w'} - \langle \sum_i w_i a_i \rangle_{w'} = \langle cov(w', a) \rangle_{w'} - \langle cov(w, a) \rangle_{w'} = \langle cov(w', a) \rangle_{w'} - cov(w, a)\,. \tag{22}$$

If the covariance of the new fitness values w'_i and the probabilities of the alleles to be present a_i is on average lower than it was before the SPFL change, β is negative and the current allele fitness on average decreases when the SPFL changes.

We should first prove the existence of a positive correlation between the fitness of an allele and its probability.

If time is viewed as a discrete variable, then the substitution matrix f represents the probability of the allele j to be replaced by the allele i at the next time step:

$$f = \frac{1}{N}\begin{bmatrix} N - \sum_{i\neq 1} f_{i1} & f_{12} & f_{13} & \cdots & f_{1N} \\ f_{21} & N - \sum_{i\neq 2} f_{i2} & f_{23} & \cdots & f_{2N} \\ f_{31} & f_{32} & N - \sum_{i\neq 3} f_{i3} & \cdots & f_{3N} \\ \vdots & \vdots & \vdots & \ddots & \vdots \\ f_{N1} & f_{N2} & f_{N3} & \cdots & N - \sum_{i\neq n} f_{iN} \end{bmatrix}. \tag{23}$$

Here, $f(s_{ij}) = f_{ij}$ is the monotonically increasing function of selection coefficient. We shall prove that under selection, the correlation between fitness vector w_i and the distribution of allele probabilities a_i emerges. For this purpose, we will demonstrate that the frequency distribution at the next time step a'_i will be on average more strongly correlated with w_i than the initial a_i:

$$\Delta = \langle cov(w, a') \rangle_a - \langle cov(w, a) \rangle_a \geq 0\,. \tag{24}$$

Based on equations 1 and 2,

$$cov(w,a) = \sum_k w_k a_k - (\sum_k w_k)(\sum_k a_k) = \sum_k w_k a_k - 1\,. \tag{25}$$

We can substitute this expression into the equation 24:

$$\Delta = \langle cov(w, a') \rangle_a - \langle cov(w, a) \rangle_a = \langle \sum_k w_k a'_k \rangle_a - \langle \sum_k w_k a_k \rangle_a\,. \tag{26}$$

The frequency of the allele k at the next time step a'_k is the result of multiplying the matrix f by a:

$$a'_k = \sum_j f_{kj} a_j \,. \tag{27}$$

Then

$$\begin{aligned} \Delta = \langle \sum_k w_k a'_k \rangle_a - \langle \sum_k w_k a_k \rangle_a = \\ \langle \sum_k w_k \sum_j f_{kj} a_j \rangle_a - \langle \sum_k w_k a_k \rangle_a = \langle \sum_k w_k \sum_j f_{kj} a_j - \sum_k w_k a_k \rangle_a = \\ \langle \sum_k w_k (\sum_j f_{kj} a_j - a_k) \rangle_a = \langle \sum_k w_k (\sum_j f_{kj} a_j - \sum_j I_{kj} a_j) \rangle_a = \\ \langle \sum_k w_k (\sum_j f_{kj} - \sum_j I_{kj}) a_j) \rangle_a = \langle \sum_k w_k \sum_j (f_{kj} - I_{kj}) a_j \rangle_a = \\ \sum_k w_k \sum_j (f_{kj} - I_{kj}) \langle a_j \rangle_a = \sum_k w_k \sum_j (f_{kj} - I_{kj}))(1/N), \end{aligned} \tag{28}$$

where I is the identity matrix. Therefore, since $N > 0$, the inequality 24 is transformed to

$$\sum_k w_k \sum_j (f_{kj} - I_{kj}) \geq 0 \,. \tag{29}$$

The matrix in the brackets is as follows:

$$f_{ij} - I_{ij} = \frac{1}{N} \begin{bmatrix} -\sum_{i\neq 1} f_{i1} & f_{12} & f_{13} & \dots & f_{1N} \\ f_{21} & -\sum_{i\neq 2} f_{i2} & f_{13} & \dots & f_{2N} \\ f_{31} & f_{23} & -\sum_{i\neq 3} f_{i3} & \dots & f_{3N} \\ \vdots & \vdots & \vdots & \ddots & \vdots \\ f_{N1} & f_{N2} & f_{N3} & \dots & -\sum_{i\neq n} f_{iN} \end{bmatrix} \tag{30}$$

The inner sum in the equation 29 is the sum of the kth row of this matrix:

$$\begin{aligned} \sum_k w_k \sum_j (f_{kj} - I_{kj}) = \sum_k w_k (-\sum_{i\neq k} f_{ik} + \sum_{j\neq k} f_{kj}) = \\ \sum_k w_k \sum_{i\neq k} (f_{ki} - f_{ik}) = \sum_{ki} w_k g_{ki} \,, \end{aligned} \tag{31}$$

where we denoted the $f_{ki} - f_{ik}$ matrix as g_{ki}. The g_{ki} matrix is anti-symmetrical, i. e. $g_{ki} = -g_{ik}$:

$$g_{ki} = f_{ki} - f_{ik} = -(f_{ik} - f_{ki}) = -g_{ik} \,. \tag{32}$$

Therefore, the diagonal values of the g_{ki} matrix are zero.

The equation 31 is the sum of the g_{ki} matrix values with some weights w_k. In the trivial case, if the weights are equal, the sum of the anti-symmetrical matrix values will be zero. However, if it's not the case (i. e. the fitness values of the alleles are not equal and the SPFL is not "flat"), the sum will be not equal to zero and, moreover, positive. To prove it, we split the equation 31 by the g_{ki} matrix diagonal:

$$\begin{aligned} \sum_{ki} w_k g_{ki} = \sum_{ki,k<i} w_k g_{ki} + \sum_{ki,k>i} w_k g_{ki} = \sum_{ki,k<i} w_k g_{ki} + \sum_{ik,i>k} w_i g_{ik} = \\ \sum_{ki,k<i} (w_k g_{ki} + w_i g_{ik}) = \sum_{ki,k<i} (w_k g_{ki} - w_i g_{ki}) = \sum_{ki,k<i} g_{ki}(w_k - w_i) \,. \end{aligned} \tag{33}$$

Assume that the fitness vector is sorted highest to lowest:

$$w_1 \geq w_2 \geq w_3 \geq \cdots \geq w_N \,. \tag{34}$$

Then, for each k, the selection coefficients s_{ki} and the probabilities of the $k \longrightarrow i$ substitution $f(s_{kj}) = f_{kj}$ are also sorted in a decreasing order:

$$\begin{aligned} s_{k1} \geq s_{k2} \geq s_{k3} \geq \cdots \geq s_{kN}\,, \\ f_{k1} \geq f_{k2} \geq f_{k3} \geq \cdots \geq f_{kN}\,. \end{aligned} \tag{35}$$

The sum in the equation 33 is taken over the upper triangular portion of the g_{ki} matrix and, based on the decreasing order of the alleles fitnesses (equation 35), contains only the positive sums:

$$k < i \Rightarrow w_k \geq w_i \Rightarrow (w_k - w_i) \geq 0 \tag{36}$$

$$k < i \Rightarrow w_k \geq w_i \Rightarrow s_{ki} \geq s_{ik} \Rightarrow f_{ki} \geq f_{ik} \Rightarrow g_{ki} = f_{ki} - f_{ik} \geq 0\,. \tag{37}$$

If there is at least one pair of alleles i and k such that $w_i \neq w_k$, the sum is positive:

$$\sum_{ki,k<i} g_{ki}(w_k - w_i) > 0 \tag{38}$$

From equation 28, Δ is also positive. In other words, even a single substitution increases the correlation between the fitness of the allele and the probability that it resides at the site; random SPFL changes on average eliminate this correlation, decreasing, on average, the fitness of the current allele ($\beta < 0$).

3. Generalization of the frequent SPFL changes case ($\tau_M \approx \tau_L$) accounting for differences in mutation rates μ_{ij}

In the above, we assumed that mutations between different alleles are equiprobable, so that the relative rates of substitutions are only dependent on the corresponding selection coefficients (eqn. 23). If there are differences in the probabilities of $i \longrightarrow j$ mutations (μ_{ij}), the substitution matrix f (eqn. 23) will be transformed:

$$f = \frac{1}{N}\begin{bmatrix} N - \sum_{i\neq 1} f_{i1}\mu_{i1} & f_{12}\mu_{12} & f_{13}\mu_{13} & \cdots & f_{1N}\mu_{1N} \\ f_{21}\mu_{21} & N - \sum_{i\neq 2} f_{i2}\mu_{i2} & f_{13}\mu_{13} & \cdots & f_{2N}\mu_{2N} \\ f_{31}\mu_{31} & f_{32}\mu_{32} & N - \sum_{i\neq 3} f_{i3}\mu_{i3} & \cdots & f_{3N}\mu_{3N} \\ \vdots & \vdots & \vdots & \ddots & \vdots \\ f_{N1}\mu_{N2} & f_{N2}\mu_{N2} & f_{N3}\mu_{N3} & \cdots & N - \sum_{i\neq n} f_{iN}\mu_{iN} \end{bmatrix}. \tag{39}$$

Accordingly, the g_{ki} matrix from eqn. 31 will be equal to $f_{ki}\mu_{ki} - f_{ik}\mu_{ik}$. The g_{ki} matrix will still be anti-symmetrical, i. e. $g_{ki} = -g_{ik}$. However, the g_{ki} will no longer necessarily be positive: it may be negative if the mutation bias and selection act in opposite directions. Indeed, the probability that a site is occupied, at equilibrium, by a deleterious allele can be high if the rate of mutation to this allele is much higher than the rate of mutation from this allele [2, 3].

Nevertheless, we'll prove that the eqn. 38 will still hold when we average across all possible new SPFLs, i. e.

$$\langle \sum_{ki,k<i} g_{ki}(w_k - w_i) \rangle_w > 0, \tag{40}$$

as long as the selection coefficient (corresponding to the new SPFL) and the mutation rate for a pair of alleles are independent of each other:

$$\langle f_{ik}\mu_{ik} \rangle_w = \langle f_{ik} \rangle_w \langle \mu_{ik} \rangle_w = \langle f_{ik} \rangle_w \mu_{ik}\,. \tag{41}$$

Equation 40 can be transformed:

$$
\begin{aligned}
n\langle\Delta\rangle_w &= \langle \sum_{ki,k<i} g_{ki}(w_k - w_i)\rangle_w = \langle \sum_{ki,k<i} (f_{ki}\mu_{ki} - f_{ik}\mu_{ik})(w_k - w_i)\rangle_w = \\
\sum_{ki,k<i} \langle (f_{ki}\mu_{ki} - f_{ik}\mu_{ik})(w_k - w_i)\rangle_w &= \sum_{ki,k<i} \langle f_{ki}\mu_{ki}(w_k - w_i)\rangle_w - \sum_{ki,k<i} \langle f_{ik}\mu_{ik}(w_k - w_i)\rangle_w = \\
&\sum_{ki,k<i} \langle f_{ki}\mu_{ki}(w_k - w_i)\rangle_w + \sum_{ki,k>i} \langle f_{ki}\mu_{ki}(w_k - w_i)\rangle_w = \\
&\sum_{ki} \langle f_{ki}\mu_{ki}(w_k - w_i)\rangle_w = \sum_{ki} \mu_{ki} \langle f_{ki}(w_k - w_i)\rangle_w \,.
\end{aligned} \tag{42}
$$

Since f_{ki} is a monotonic function of $(w_k - w_i)$, they are positively correlated:

$$
\langle f_{ki}(w_k - w_i)\rangle_w - \langle f_{ki}\rangle_w \langle (w_k - w_i)\rangle_w = \langle f_{ki}(w_k - w_i)\rangle_w - 0 = \delta > 0\,. \tag{43}
$$

Then, based on eqn. 42,

$$
n\langle\Delta\rangle_w = \sum_{ki} \mu_{ki}\delta = \delta \sum_{ki} \mu_{ki}\,. \tag{44}
$$

Since the mutation rates are also positive,

$$
n\langle\Delta\rangle_w = \delta \sum_{ki} \mu_{ki} > 0\,. \tag{45}
$$

Therefore, we have proven that given any mutation rate matrix, even a single substitution on average (across all possible SPFLs) increases the correlation between the frequency of the allele and its fitness, assuming that selection preferences are independent of mutation biases.

Supplementary Note 2

The rate of evolution under fluctuating selection

According to the diffusion fluctuation theory [4–6], if the fluctuations in the SPFL are very rapid, the resulting landscape will be "quasi-neutral". In this case, the substitution rate will be reduced, and not increased, by further increase in the fluctuation rate, ultimately reaching the neutral value. This is not modeled in the Markov chain based approach we used for simulations [7]. We assume that most fluctuation-induced substitutions occur when the SPFL change frequency is lower than the rate of evolution or comparable to it, so our model is still suitable to study evolution under fluctuating selection.

Supplementary Note 3

Senescence and positive selection

While senescence underlies positive selection in evolution of vertebrates and insects (Fig. 5b,d), these phenomena are distinct. Indeed, firstly, weak senescence of a highly beneficial allele currently occupying an amino acid site can result in weakening of the negative selection favoring it; however, if this allele remains the optimal one, the direction of selection remains the same, and no positive selection in favor of a different allele starts to act (see examples in Fig. 2b and c, right panel). Secondly, there are models of positive selection that don't imply senescence, including variations of "stairway to heaven" (STH) landscapes [8–10]. Importantly, the existing models of positive selection do not imply senescence, and senescence is not observed in them, as evidenced by the simulated datasets with positive selection and no senescence (Fig. 5a,c).

Senescence and entrenchment in evolution of amino acid sites

Supplementary Figures

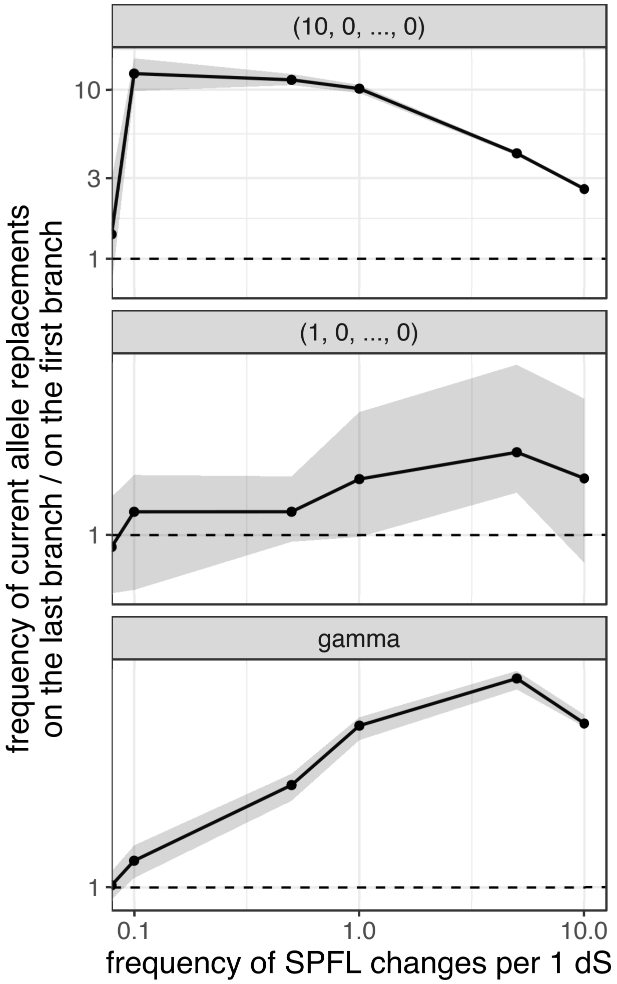

Supplementary Figure 1 **Random SPFL changes lead to the increase of the rate of the current allele replacements with the time since its origin**

For the static SPFLs of different shapes, i.e. if initial fitness values are drawn from some distribution and don't change with time, the frequency of the replacements of the current allele remain the same for "early" and "late" branches. If fitness values are redrawn from the same distribution with some frequency (i. e. SPFL changes randomly with time), the rate of current allele replacements on the "late" branch increases. The mean values and 95% confidence bands obtained in 10 simulation repeats are shown.

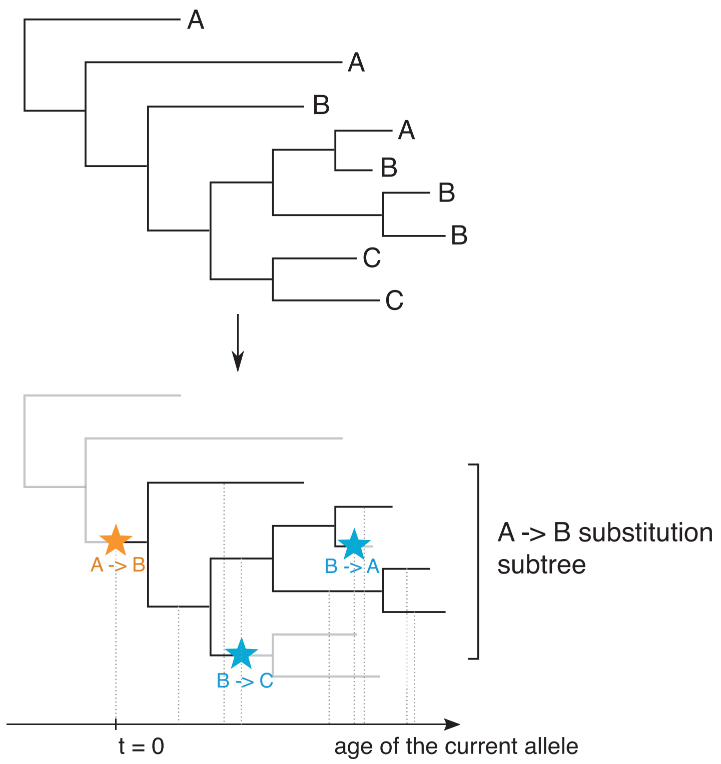

Supplementary Figure 2 **The concept of substitution subtrees**
For every individual amino acid site, ancestral state reconstruction can be used to infer allele substitution history at this site. For every replacement $A \rightarrow B$ (orange) on any internal branch of the phylogeny, we can define the corresponding substitution subtree — the contiguous segment of the phylogeny where every internal node carries the derived variant B. Within this substitution subtree, the current allele B can be replaced by the ancestral variant A ($B \rightarrow A$ substitution) or some other variant C ($B \rightarrow C$ substitution) in the course of allele losses (blue). For such subtree, A is the ancestral allele and B is the current allele.

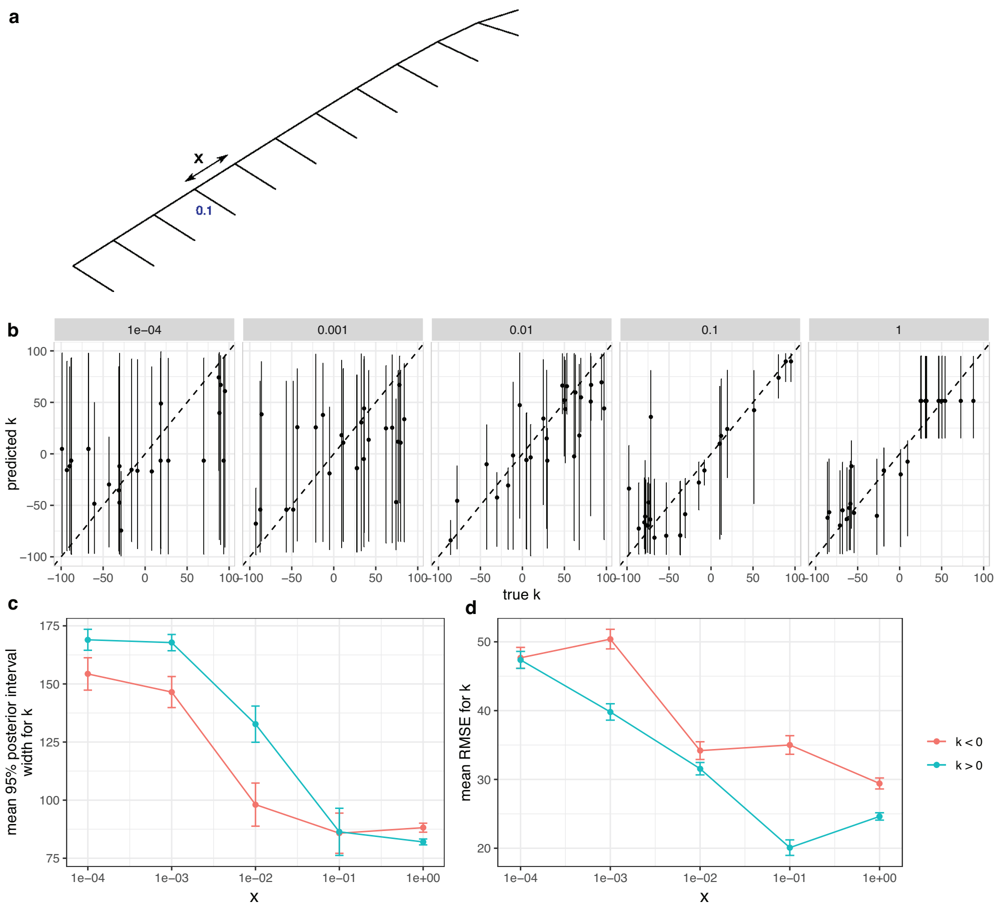

Supplementary Figure 3 **Performance of ABC inference of k on phylogenies of different shape**

a, The phylogenetic tree used for ABC performance validation. The topology of the phylogeny and the length of the terminal branches (0.1 dS) remained the same, while the length of the internal branches (x) varied from 1e-4 ("star-like" tree) to 1. **b**, The 95% posterior probability intervals for k estimated on the phylogenies with varying x; the points show the median of the posterioir probability distribution. **c**, average width of the 95% posterior probability intervals for k and **d**, average RMSE of the prediction on the phylogenies with varying x (data are presented as mean values ± SEM based on 25 simulations).

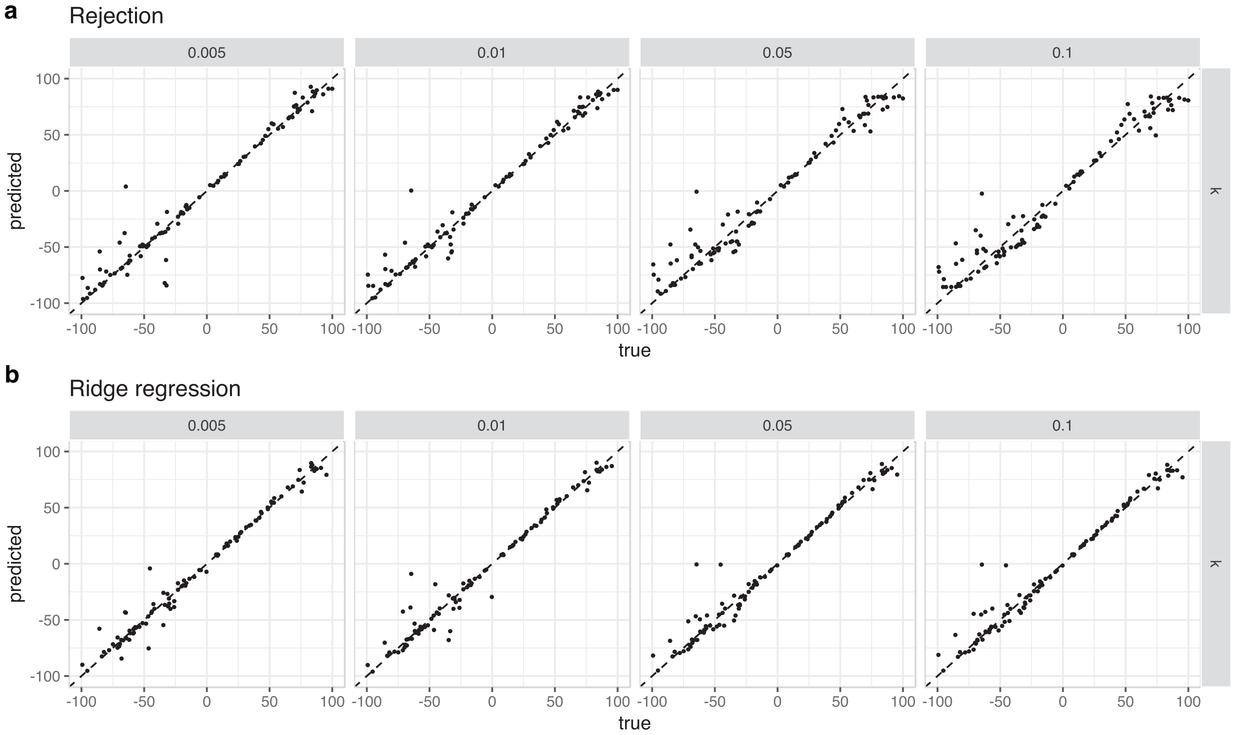

Supplementary Figure 4 **Accuracy of the prediction for the two-parameter model**
Estimation accuracy for the rate of the change of the current allele fitness k under 0.005, 0.01, 0.05 and 0.1 acceptance threshold using simple rejection ABC (**a**) and ABC with local Ridge regression adjustment (**b**). The average prediction errors are listed in Supplementary Table 3.

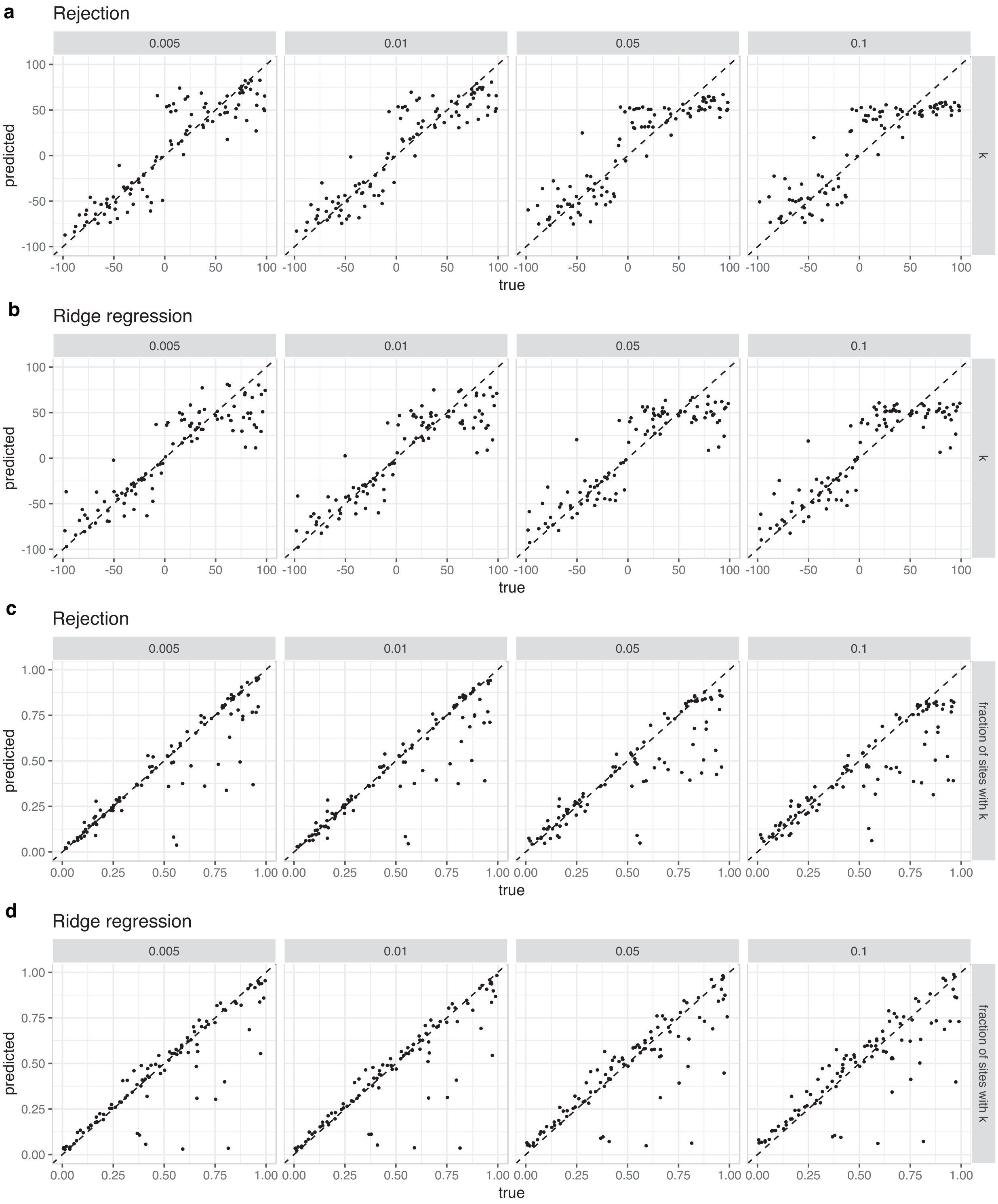

Supplementary Figure 5 **Accuracy of the prediction for the three-parameter model**
Estimation accuracy for the rate of the change of the current allele fitness k **(a,b)** and the fraction of alleles with changing fitness **(c,d)** under 0.005, 0.01, 0.05 and 0.1 acceptance threshold using simple rejection ABC **(a,c)** and ABC with local Ridge regression adjustment **(b,d)**. The average prediction errors are listed in Supplementary Table 3.

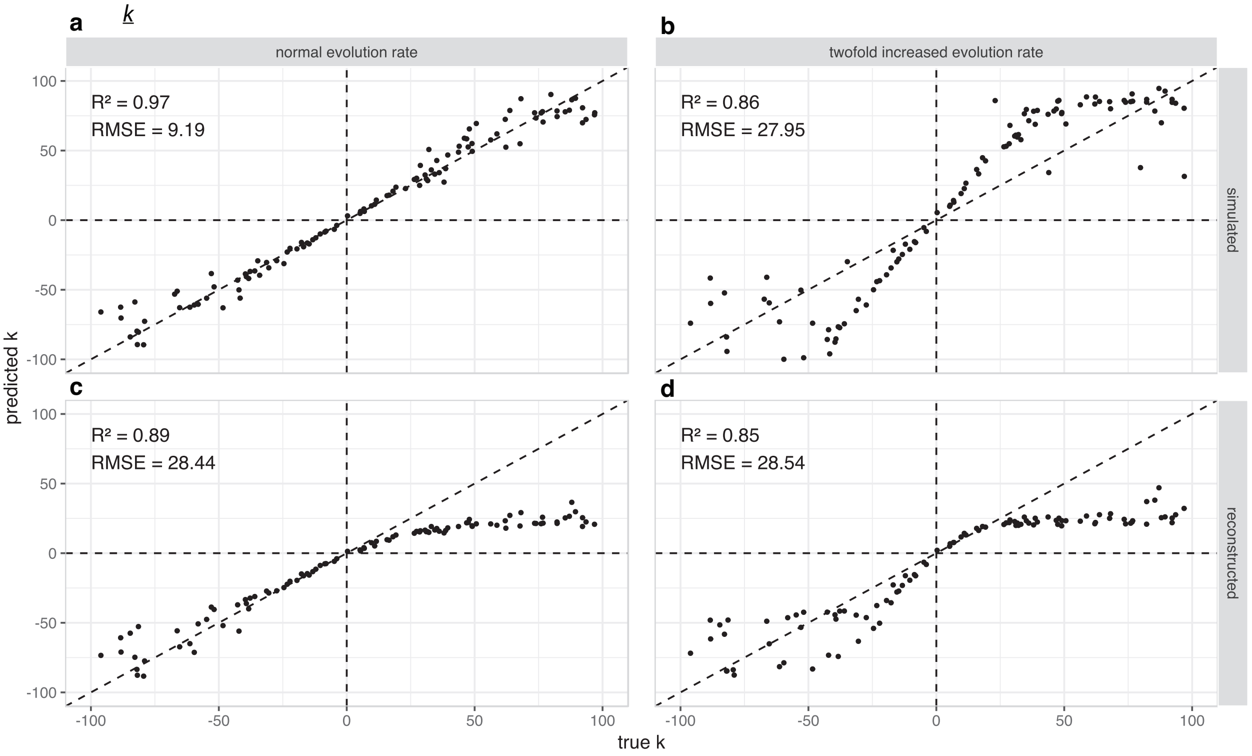

Supplementary Figure 6 **Accuracy of the prediction for the two-parameter model under accelerated evolution rate and reconstruction of ancestral states**

The rate of the current allele fitness change k was estimated on simulations with normal (**a,c**) and twofold increased overall substitution rate (**b,d**). For **a,b** the substitution history was taken directly from simulations; for **c,d** it was reconstructed with *codeml*. The confusion matrices for all four cases are shown in Supplementary Table 2.

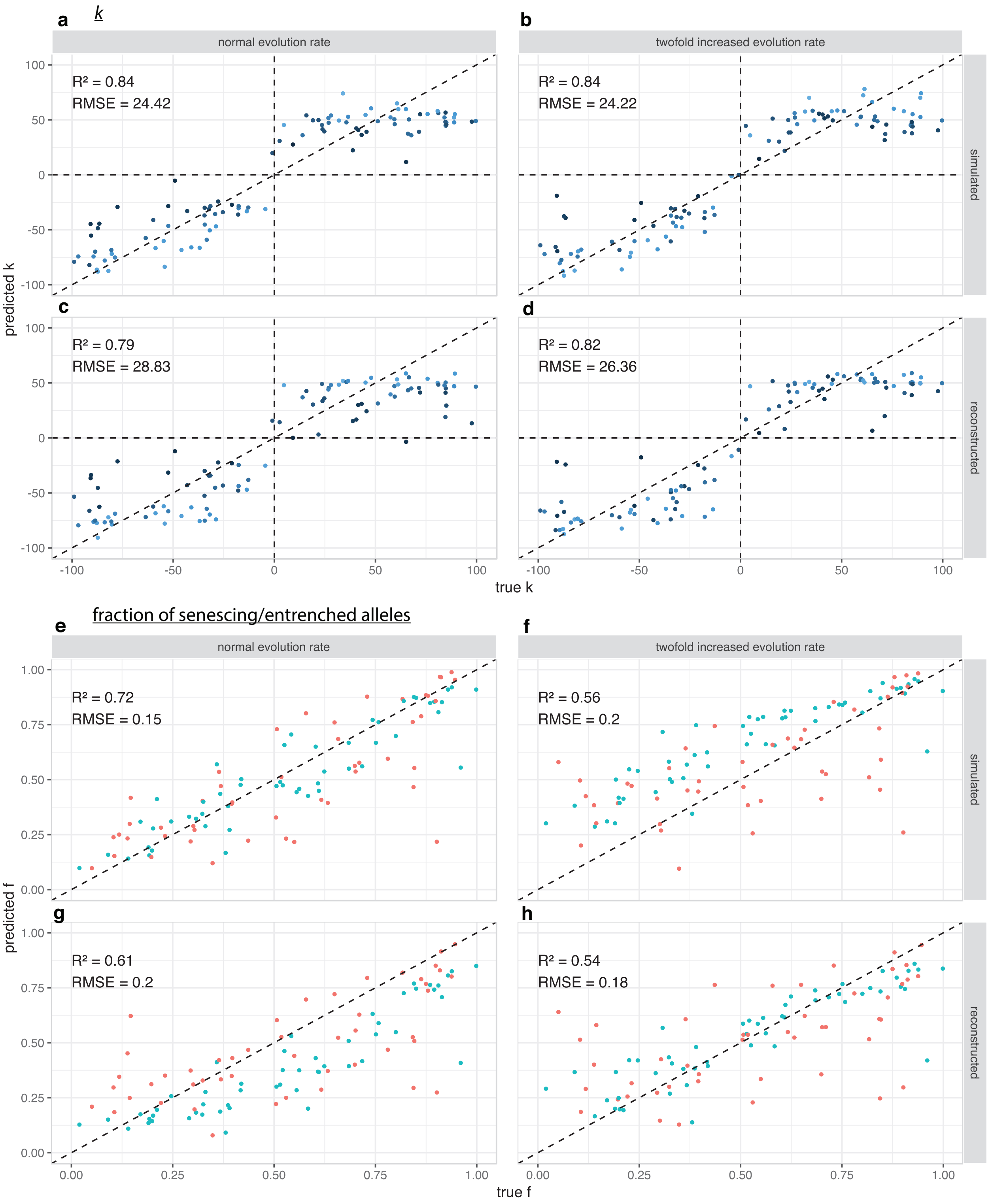

Supplementary Figure 7 **Accuracy of ABC prediction for the three-parameter model with and without accelerated evolution rate and reconstruction of ancestral states**

a-d, The rate of the current allele fitness change k was estimated on simulations with normal (**a,c**) and twofold increased overall substitution rate (**b,d**). For **a,b** the substitution history was taken directly from simulations; for **c,d** it was reconstructed with *codeml*. The confusion matrices for all four cases are shown in Supplementary Table 2. **e-h**, The same for the fraction of alleles with changing fitness based on the same set of simulations. Blue dots correspond to the simulations with $k > 0$ (entrenchment), red dots correspond to the simulations with $k < 0$ (senescence).

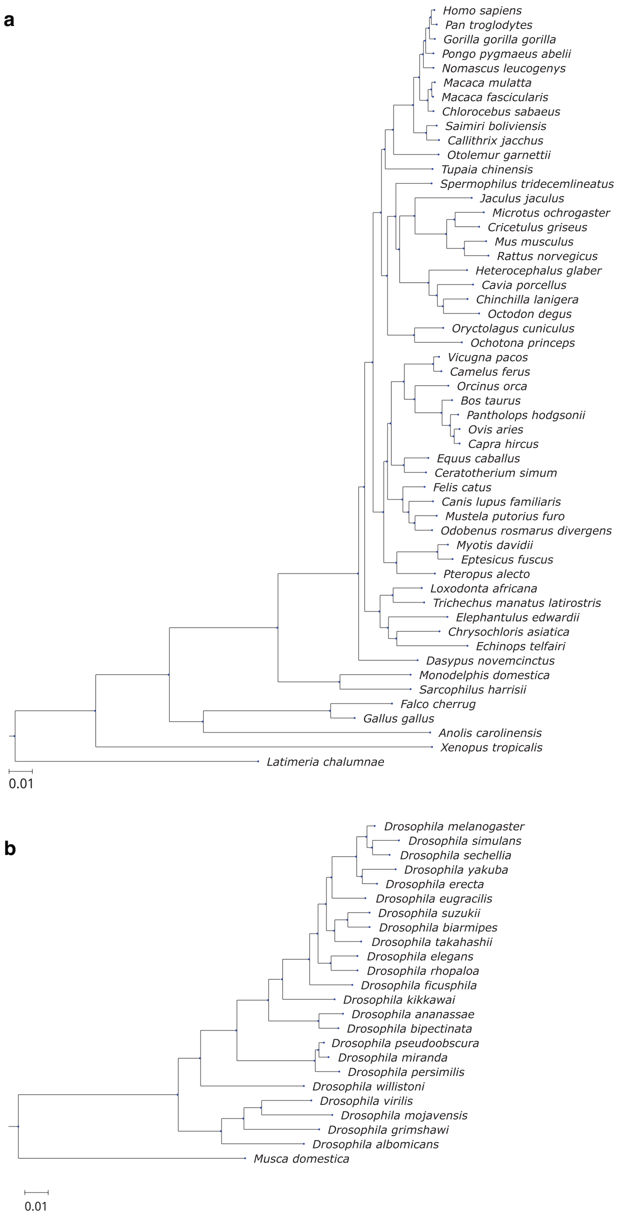

Supplementary Figure 8 **The phylogenies used for the inference of current allele fitness change**
The phylogenies of 53 species of vertebrates (**a**) and of 24 species of insects (**b**) from UCSC Genome Browser database. The branch lengths are given in dS.

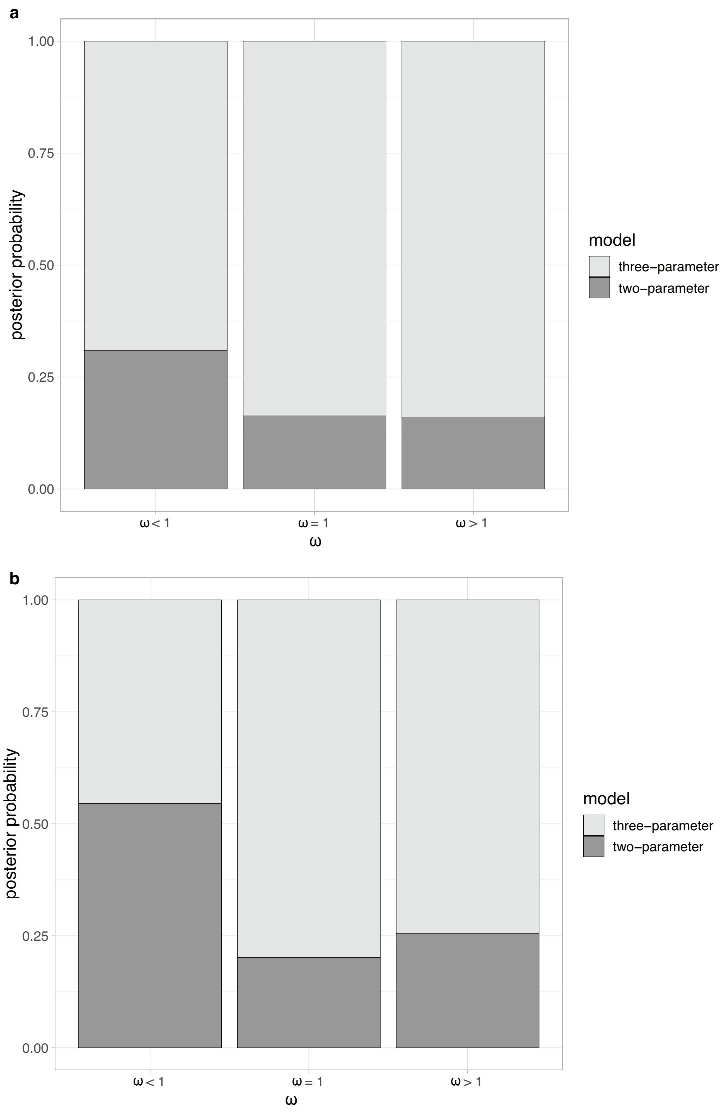

Supplementary Figure 9 **The posterior probabilities for the two- and three-parameter models**
The ABC posterior probabilities for the two-parameter (dark grey) and the three-parameter (light grey) models for the vertebrates (**a**) and insects (**b**) data. For all datasets, except sites with $\omega < 1$ of insects, the three-parameter model is preferable.

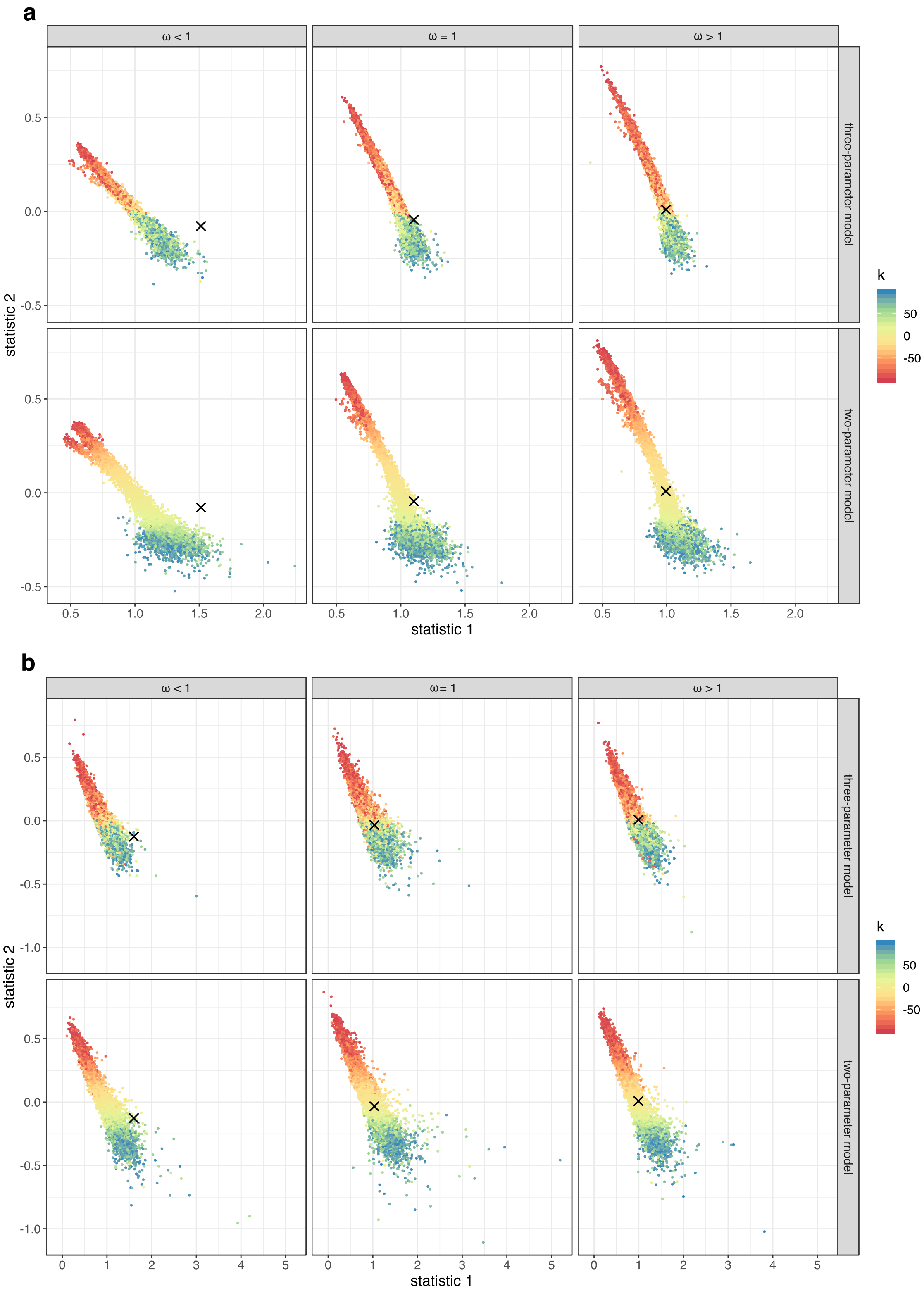

Supplementary Figure 10 **The distribution of summary statistics in simulations and data**
Each dot represents one simulation in the space of two summary statistics used in ABC parameter inference. The color shows the value of k used in the simulation (blue corresponds to entrenchment ($k > 0$), red corresponds to senescence ($k < 0$). The distributions for both the two-parameter and the three-parameter models are shown. The crosses show the summary statistics calculated from protein sequences of vertebrates (**a**) and insects (**b**).

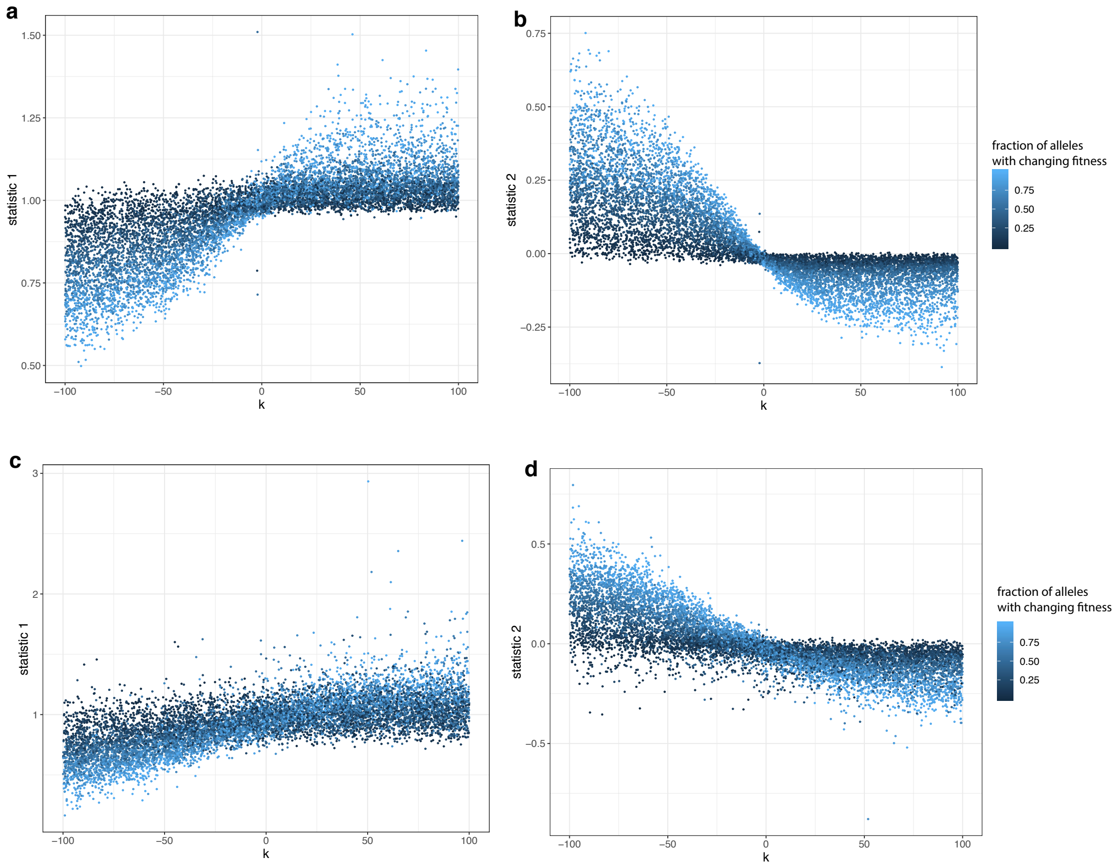

Supplementary Figure 11 **The dependence of the summary statistics on the simulation parameters**
The simulations were performed on the phylogenies of vertebrates (**a,b**) and insects (**c,d**). Each dot represents one simulation with some parameters k and fraction of alleles with log fitness linearly changing with the rate k (color). For both phylogenies, summary statistics reflect both the direction and the rate of current allele fitness change k and the fraction of alleles with dynamic fitness.

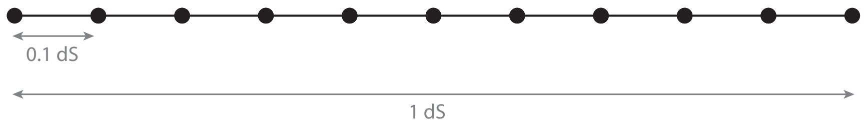

Supplementary Figure 12 **The model phylogeny used in simulations**
The phylogeny which was used to demonstrate the effects of senescence, entrenchment and the heterogeneity of the substitution rates.

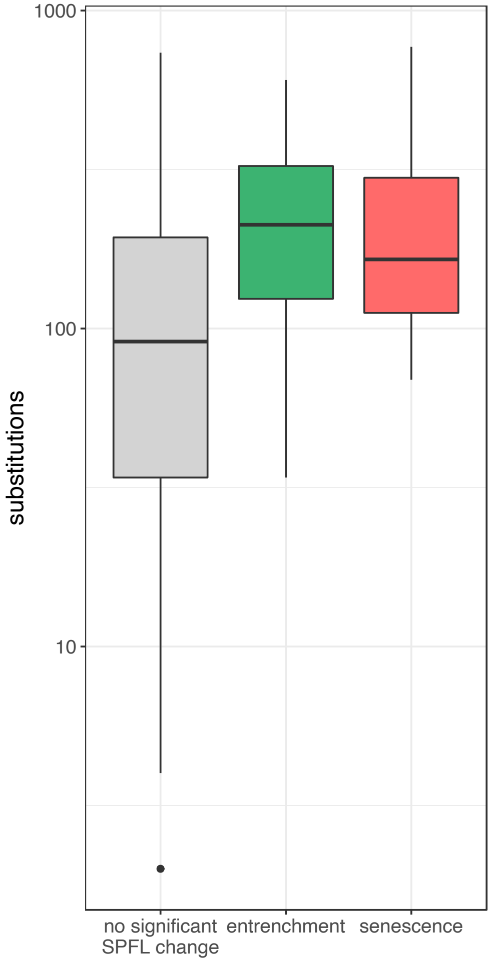

Supplementary Figure 13 **Distribution of substitution rates in mitochondrial sites** The distribution of number of substitutions in mitochondrial sites annotated as senescing (red, n=21) or entrenched (green, n=28) and in the sites where no significant SPFL changes were detected (gray, n=791). Box bounds correspond to the 25% and 75% percentiles, the centre shows the median; whiskers show the corresponding percentile ± 1.5IQR; the dot corresponds to an outlier.

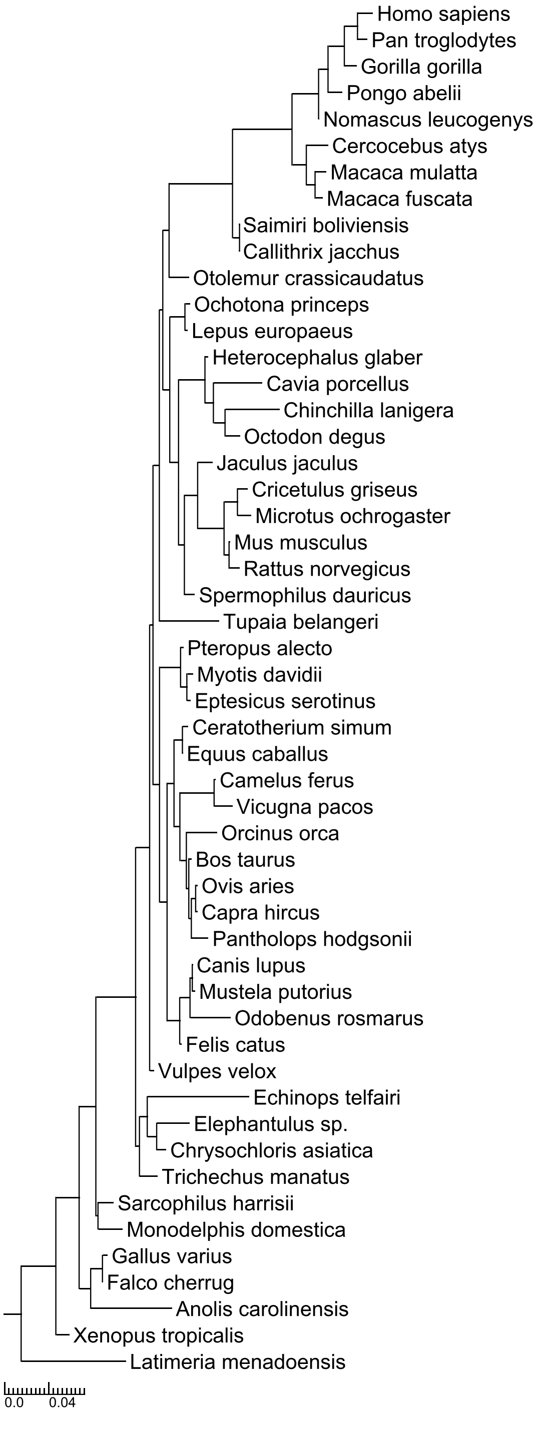

Supplementary Figure 14 **The mitochondrial phylogeny of vertebrates used for ABC validation**
The phylogeny which was used to demonstrate the robustness of our method to the errors in tree shape and branch lengths.

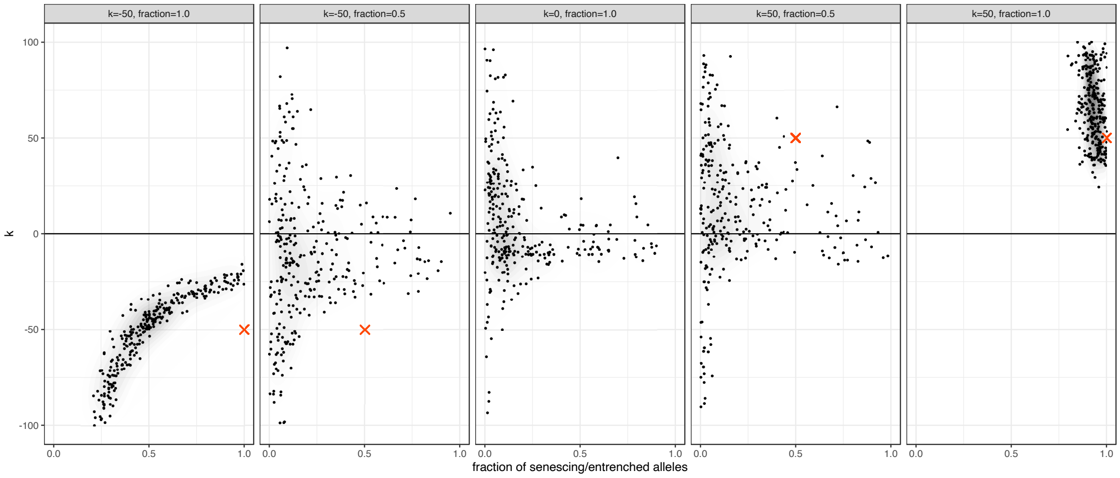

Supplementary Figure 15 **Results of ABC under wrong assumption of the tree shape**
Black dots – the parameters of accepted simulations, red crosses – the true parameters for each simulated dataset. For the static SPFL dataset (middle panel) k was equal to zero for all alleles. The prior simulations used the whole-genome phylogeny of vertebrates, while the data were simulated on the mitochondrial tree.

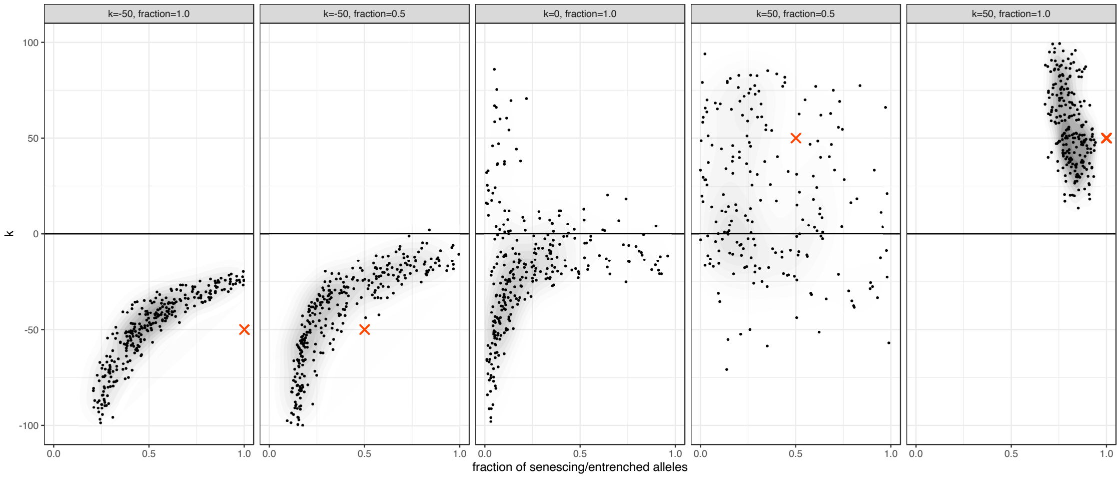

Supplementary Figure 16 **Results of ABC under wrong assumption of the distribution of fitness values**
Black dots – the parameters of accepted simulations, red crosses – the true parameters for each simulated dataset. For the static SPFL dataset (middle panel) k was equal to zero for all alleles. The fitness values for alleles were driven from the gamma distribution in the prior simulations and from the lognormal distribution for simulated data.

Supplementary Table 1

Senescence and entrenchment in mitochondrial genes of Metazoa. The table shows mitochondrial sites which show statistically significant entrenchment (regression coefficient < 0) or senescence (regression coefficient > 0). Regression coefficients are calculated using binomial logistic regression (for more details, see main text).

AC (human)	protein name	position	p-value	regression coefficient	q-value
P00846.1	ATP6	5	9.25E-07	-29.13	5.91E-04
P00846.1	ATP6	10	8.40E-07	11.30	5.75E-04
P00846.1	ATP6	26	3.86E-07	-10.14	3.08E-04
P00846.1	ATP6	69	1.25E-04	-37.11	3.14E-02
P00846.1	ATP6	70	6.59E-05	-16.23	1.97E-02
P00846.1	ATP6	71	4.45E-14	-9.02	2.13E-10
P00846.1	ATP6	80	2.44E-08	7.34	3.34E-05
P00846.1	ATP6	105	5.21E-06	9.09	2.27E-03
P00846.1	ATP6	111	4.72E-08	7.73	5.65E-05
P00846.1	ATP6	116	2.09E-04	-8.38	4.17E-02
P00846.1	ATP6	125	1.46E-08	6.34	2.81E-05
P00846.1	ATP6	141	1.86E-08	5.95	2.98E-05
P00846.1	ATP6	178	1.47E-04	-7.60	3.61E-02
P00846.1	ATP6	179	1.72E-09	4.23	5.49E-06
P00846.1	ATP6	224	1.89E-04	-10.28	3.97E-02
P00395.1	COX1	52	6.69E-06	6.37	2.71E-03
P00395.1	COX1	132	2.30E-06	-8.60	1.22E-03
P00395.1	COX1	185	1.88E-05	-16.80	6.44E-03
P00395.1	COX1	256	2.10E-05	-30.61	6.93E-03
P00395.1	COX1	336	6.79E-06	-13.88	2.71E-03
P00395.1	COX1	389	1.07E-04	-12.40	2.86E-02
P00395.1	COX1	399	1.71E-06	-30.26	9.61E-04
P00395.1	COX1	414	3.58E-06	-8.05	1.63E-03
P00395.1	COX1	415	2.58E-06	-9.15	1.30E-03
P00395.1	COX1	448	7.98E-05	-14.43	2.25E-02
P00395.1	COX1	491	1.92E-04	-37.47	3.97E-02
P00403.1	COX2	21	1.75E-04	41.65	3.97E-02
P00403.1	COX2	37	1.03E-07	6.56	1.10E-04
P00403.1	COX2	51	2.74E-05	30.72	8.48E-03
P00403.1	COX2	56	3.91E-18	12.63	3.74E-14
P00403.1	COX2	57	1.85E-05	-28.14	6.44E-03
P00403.1	COX2	112	2.28E-04	35.40	4.47E-02
P00403.1	COX2	217	2.40E-09	6.28	5.75E-06
P00414.2	COX3	47	1.52E-04	10.36	3.64E-02
P00414.2	COX3	60	1.18E-04	3.37	3.05E-02
P00414.2	COX3	166	7.85E-07	-9.71	5.75E-04
P00156.2	CYTB	4	3.42E-06	-4.17	1.63E-03
P00156.2	CYTB	10	1.93E-04	-51.60	3.97E-02
P00156.2	CYTB	38	1.95E-04	16.61	3.97E-02

P00156.2	CYTB	81	1.10E-05	-59.64	4.21E-03
P00156.2	CYTB	93	1.90E-04	-17.48	3.97E-02
P00156.2	CYTB	121	2.17E-07	28.84	1.89E-04
P00156.2	CYTB	180	1.35E-06	8.57	8.07E-04
P00156.2	CYTB	183	7.39E-05	14.84	2.15E-02
P00156.2	CYTB	218	1.83E-05	-23.65	6.44E-03
P00156.2	CYTB	225	8.90E-05	9.37	2.44E-02
P00156.2	CYTB	235	1.70E-07	-42.63	1.63E-04
P00156.2	CYTB	337	1.89E-04	-20.53	3.97E-02
P00156.2	CYTB	346	2.65E-05	-4.01	8.47E-03

Supplementary Table 2

Confusion matrices for ABC-based mehod for detecting senescence and entrenchment. The tables show confusion matrices for two models based on simulations of senescing (k < 0) and entrenched (k > 0) sites. The prior set of simulations used for ABC was the same, while simulations for cross-validation were perfomed under normal or twofold accelerated evolution rate; we also tested whether reconstuction of ancestral states with *codeml* changes the predictions. For the false presictions for the three-parameter model (three cases; shown on orange background), the values of *k* and the fraction of senescing/entrenched alleles are provided.

Two-parameter model

Normal evolution rate, true ancestral states

		Predicted	
		k > 0	k < 0
True	k > 0	56	0
	k < 0	0	44

Accelerated evolution rate (x2), true ancestral states

		Predicted	
		k > 0	k < 0
True	k > 0	56	0
	k < 0	0	44

Normal evolution rate, reconstructed ancestral states

		Predicted	
		k > 0	k < 0
True	k > 0	56	0
	k < 0	0	44

Accelerated evolution rate (x2), reconstructed ancestral states

		Predicted	
		k > 0	k < 0
True	k > 0	56	0
	k < 0	0	44

Three-parameter model

Normal evolution rate, true ancestral states

		Predicted		False predictions
		k > 0	k < 0	
True	k > 0	52	0	
	k < 0	1	47	k = -0.95; fraction = 0.35

Accelerated evolution rate (x2), true ancestral states

		Predicted		
		k > 0	k < 0	
True	k > 0	52	0	
	k < 0	1	47	k = -0.95; fraction = 0.35

Normal evolution rate, reconstructed ancestral states

		Predicted		
		k > 0	k < 0	
True	k > 0	51	1	k = 65.15; fraction = 0.09
	k < 0	0	48	

Accelerated evolution rate (x2), reconstructed ancestral states

		Predicted	
		k > 0	k < 0
True	k > 0	52	0
	k < 0	0	48

Supplementary Table 3

Cross-validation of ABC. We used cross-validation to determine the tolerance rate (the fraction of simulations accepted) and the algorithm giving the smallest prediction error for *k* (and, for the three-parameter model, the fraction of sites under *k*). For vertebrates and insects datasets, we used local ridge regression with tolerance rate 1% (shown on yellow background).

Two-parameter model

method	tolerance rate	prediction error	
		k	log10(alpha)
rejection	0.005	0.029	0.818
	0.01	0.027	0.801
	0.05	0.029	0.937
	0.1	0.036	0.981
local ridge regression	0.005	0.034	1.164
	0.01	0.030	1.047
	0.05	0.028	0.952
	0.1	0.029	0.971

Three-parameter model

method	tolerance rate	prediction error		
		k	fraction of sites under k	log10(alpha)
rejection	0.005	0.232	0.580	0.870
	0.01	0.225	0.581	0.854
	0.05	0.233	0.597	0.893
	0.1	0.242	0.596	0.931
local ridge regression	0.005	0.223	0.333	0.829
	0.01	0.229	0.330	0.784
	0.05	0.234	0.312	0.784
	0.1	0.241	0.313	0.802

Supplementary References

[1] Kimura, M., "On the probability of fixation of mutant genes in a population," *Genetics*, Vol. 47, 1962, pp. 713–719.

[2] Kondrashov, A. S., and Crow, J. F., "A molecular approach to estimating the human deleterious mutation rate," *Hum. Mutat.*, Vol. 2, No. 3, 1993, pp. 229–234.

[3] Eyre-Walker, A., and Keightley, P. D., "High genomic deleterious mutation rates in hominids," *Nature*, Vol. 397, No. 6717, 1999, pp. 344–347.

[4] Kimura, M., "Process Leading to Quasi-Fixation of Genes in Natural Populations Due to Random Fluctuation of Selection Intensities," *Genetics*, Vol. 39, No. 3, 1954, pp. 280–295.

[5] Takahata, N., Ishii, K., and Matsuda, H., "Effect of temporal fluctuation of selection coefficient on gene frequency in a population," *Proc. Natl. Acad. Sci. U.S.A.*, Vol. 72, No. 11, 1975, pp. 4541–4545.

[6] Mustonen, V., and Lassig, M., "Adaptations to fluctuating selection in Drosophila," *Proc. Natl. Acad. Sci. U.S.A.*, Vol. 104, No. 7, 2007, pp. 2277–2282.

[7] Nabieva, E., and Bazykin, G. A., "SELVa: Simulator of Evolution with Landscape Variation," *bioRxiv*, 2019. doi:10.1101/647834, URL `https://www.biorxiv.org/content/early/2019/05/25/647834`.

[8] Gerrish, P. J., and Lenski, R. E., "The fate of competing beneficial mutations in an asexual population." *Genetica*, Vol. 102-103, No. 1-6, 1998, pp. 127–44.

[9] Desai, M., and Fisher, D., "Beneficial mutation–selection balance and the effect of linkage on positive selection," *Genetics*, Vol. 176, No. 3, 2007, pp. 1759–1798.

[10] Kryazhimskiy, S., Tkacik, G., and Plotkin, J. B., "The dynamics of adaptation on correlated fitness landscapes." *Proceedings of the National Academy of Sciences of the United States of America*, Vol. 106, No. 44, 2009, pp. 18638–43. doi: 10.1073/pnas.0905497106.
