# Supplementary material for: Senescence and entrenchment in evolution of amino acid sites
Source: Nat Commun. 2020 Sep 14;11:4603. doi: 10.1038/s41467-020-18366-z (PMC7490271; doi:10.1038/s41467-020-18366-z)
Supplement: Supplementary file 3 — Reporting Summary [file 41467_2020_18366_MOESM3_ESM.pdf]

nature research

Corresponding author(s): Anastasia Stolyarova

Last updated by author(s): Jul 21, 2020

Reporting Summary

Nature Research wishes to improve the reproducibility of the work that we publish. This form provides structure for consistency and transparency in reporting. For further information on Nature Research policies, see our Editorial Policies and the Editorial Policy Checklist.

Statistics

For all statistical analyses, confirm that the following items are present in the figure legend, table legend, main text, or Methods section.

n/a	Confirmed	
☐	☒	The exact sample size (n) for each experimental group/condition, given as a discrete number and unit of measurement
☐	☒	A statement on whether measurements were taken from distinct samples or whether the same sample was measured repeatedly
☐	☒	The statistical test(s) used AND whether they are one- or two-sided<br>*Only common tests should be described solely by name; describe more complex techniques in the Methods section.*
☐	☒	A description of all covariates tested
☐	☒	A description of any assumptions or corrections, such as tests of normality and adjustment for multiple comparisons
☐	☒	A full description of the statistical parameters including central tendency (e.g. means) or other basic estimates (e.g. regression coefficient) AND variation (e.g. standard deviation) or associated estimates of uncertainty (e.g. confidence intervals)
☐	☒	For null hypothesis testing, the test statistic (e.g. F, t, r) with confidence intervals, effect sizes, degrees of freedom and P value noted<br>*Give P values as exact values whenever suitable.*
☐	☒	For Bayesian analysis, information on the choice of priors and Markov chain Monte Carlo settings
☐	☒	For hierarchical and complex designs, identification of the appropriate level for tests and full reporting of outcomes
☒	☐	Estimates of effect sizes (e.g. Cohen's d, Pearson's r), indicating how they were calculated

Our web collection on statistics for biologists contains articles on many of the points above.

Software and code

Policy information about availability of computer code

Data collection	none
Data analysis	R Statistical Computing environment (v3.5.1); R packages: abc (v1.0), python (v2.7.5); python packages: numpy(v1.14.1), sklearn(v0.19.1), Bio (v1.68), ete3 (v3.1.1); codeml (part of PAML v4.8), evolver (part of PAML v4.8); SELVa (https://github.com/bazykinlab/SELVa); https://github.com/astolyarova/senescence-ABC

For manuscripts utilizing custom algorithms or software that are central to the research but not yet described in published literature, software must be made available to editors and reviewers. We strongly encourage code deposition in a community repository (e.g. GitHub). See the Nature Research guidelines for submitting code & software for further information.

Data

Policy information about availability of data

All manuscripts must include a data availability statement. This statement should provide the following information, where applicable:

- Accession codes, unique identifiers, or web links for publicly available datasets
- A list of figures that have associated raw data
- A description of any restrictions on data availability

The genomic datasets of vertebrates and insects were downloaded from UCSC database https://hgdownload.soe.ucsc.edu (Rosenbloom et al. 2015). The alignments of mitochondrial genes used in this work were published in doi: 10.1093/gbe/evx025 (Klink and Bazykin 2017). The simulated prior distributions used in the current study, the summary statistics calculated from the genomic datasets of vertebrates and insects and source code for the analysis are available at https://github.com/astolyarova/senescence-ABC.

Field-specific reporting

Please select the one below that is the best fit for your research. If you are not sure, read the appropriate sections before making your selection.

- [x] Life sciences
- [ ] Behavioural & social sciences
- [ ] Ecological, evolutionary & environmental sciences

For a reference copy of the document with all sections, see nature.com/documents/nr-reporting-summary-flat.pdf

Life sciences study design

All studies must disclose on these points even when the disclosure is negative.

Sample size	We used aligned nuclear genomes of 53 vertebrates (2,959,937 amino acid sites) and 24 insects (2,894,95 amino acid sites), and of 3,557 mitochondrial genomes of Metazoa (1,361 amino acid sites).
Data exclusions	We excluded columns of alignments with gaps for all datasets.
Replication	There are no experimental findings presented in the paper.
Randomization	No experiments were carried out.
Blinding	No allocation of groups took place.

Reporting for specific materials, systems and methods

We require information from authors about some types of materials, experimental systems and methods used in many studies. Here, indicate whether each material, system or method listed is relevant to your study. If you are not sure if a list item applies to your research, read the appropriate section before selecting a response.

Materials & experimental systems

n/a	Involved in the study	
x		Antibodies
x		Eukaryotic cell lines
x		Palaeontology and archaeology
x		Animals and other organisms
x		Human research participants
x		Clinical data
x		Dual use research of concern

Methods

n/a	Involved in the study	
x		ChIP-seq
x		Flow cytometry
x		MRI-based neuroimaging
